# Development and validation of a nomogram model integrating noninvasive detection of radial pulse wave for predicting diabetic foot risk in type 2 diabetes mellitus

**DOI:** 10.3389/fendo.2026.1758234

**Published:** 2026-02-12

**Authors:** Yuting Huo, Wen Li, Ai Xu, Siman Wang, Yuqing Wang, Jin Xu, Rui Guo, Cheng Zhao, Haixia Yan

**Affiliations:** 1 School of Traditional Chinese Medicine, Shanghai University of Traditional Chinese Medicine (TCM), Shanghai, China; 2 Department of Vascular Disease, Shanghai Traditional Chinese Medicine (TCM)- Integrated Hospital, Shanghai University of Traditional Chinese Medicine (TCM), Shanghai, China

**Keywords:** diabetic foot, nomogram, predictive model, pulse mapping parameters, type 2 diabetes mellitus

## Abstract

**Background:**

This study aimed to develop and evaluate a nomogram model based on Traditional Chinese Medicine (TCM) pulse mapping parameters to predict the risk of diabetic foot (DF) in patients with type 2 diabetes mellitus (T2DM).

**Methods:**

A retrospective analysis was performed on clinical data and pulse mapping parameters from 485 T2DM patients hospitalized in the Departments of Vascular Diseases and Endocrinology at Shanghai TCM-Integrated Hospital between July 26, 2023, and April 9, 2025. Patients were divided into a DF group (*n* = 242) and a T2DM-only group (*n* = 243) according to the presence of DF. Pulse mapping parameters and clinical indicators were compared between the two groups. Least Absolute Shrinkage and Selection Operator (LASSO) regression and multivariate logistic regression analyses were conducted to identify risk factors associated with DF development in T2DM. The risk prediction nomogram was constructed using R Studio. Model performance was evaluated using the Receiver Operating Characteristic (ROC) curve and its Area Under the Curve (AUC). Accuracy, Precision, Recall, and F1-score were calculated. Calibration curves were plotted to assess the agreement between predicted probabilities and actual outcomes, and decision curve analysis (DCA) was used to evaluate the model’s clinical utility.

**Results:**

Gender (OR = 0.501, *p* = 0.002), BMI (OR = 0.919, *p* = 0.003), hypertension (OR = 2.482, *p* < 0.001), systolic blood pressure (OR = 0.972, *p* < 0.001), blood urea nitrogen (BUN) (OR = 1.099, *p* = 0.011), and the pulse mapping parameter H5/H1 (OR = 0.002, *p* < 0.001) were identified as independent risk factors for DF in T2DM. The nomogram model based on these factors achieved an AUC of 0.786 (95% CI: 0.694–0.877) in the test set, with an Accuracy of 0.7113, Precision of 0.6481, Recall of 0.7955, and F1-score of 0.7143. The Hosmer–Lemeshow test demonstrated good calibration (χ² = 6.1672, *p* = 0.6285), showing close agreement between predicted and observed outcomes. The DCA confirmed favorable clinical applicability.

**Conclusion:**

This nomogram model provides an individualized and visualized tool for predicting DF risk in T2DM patients, offering TCM-based reference indicators for early DF risk assessment.

## 1 Introduction

Diabetes mellitus (DM) is one of the most prevalent chronic metabolic diseases worldwide and represents a major public health challenge (1). Type 2 diabetes mellitus (T2DM) accounts for over 90% of all DM cases (2). The number of people with DM has steadily increased over the past three decades, especially in China, which has one of the largest diabetic populations. Diabetic foot (DF) is one of the most serious complications of DM, characterized by foot ulcers, infections, and deep tissue destruction that can lead to amputation, severely impairing patients’ quality of life. Therefore, early diagnosis, prevention, and treatment of DF are vital for reducing the medical burden and mortality rate.

The main pathological changes in DF involve microcirculatory disorders and neurovascular damage caused by metabolic disturbances (3). Although the International Working Group on the DF (IWGDF) guidelines emphasize early risk stratification and management, most existing prediction systems rely on static indicators (e.g., ankle–brachial index, vibration perception threshold). These structure-based assessments fail to capture the critical stage when microcirculatory dysfunction progresses to tissue necrosis—the optimal window for clinical intervention.

Pulse taking is an important skill for diagnosing diseases in TCM. Objective pulse diagnosis research results enable its clinical application. Studies conducted by Yiming Hao et al. reported the noninvasive and convenient pulse-taking diagnosis has the potential to become a low-cost and accurate method to monitor the development of diabetes (4). Jiekee Lim et al. found that the new prediction model that uses pulse wave parameters and TCM clinical indices offers a non-invasive and cost-effective way to diagnose PCOS (5).

At present, the relationship between pulse wave and DF has not been reported. Current efforts to objectify and standardize TCM pulse diagnosis have achieved progress (6, 7), providing a basis for DF risk prediction model development. The nomogram model is widely applied in medical and biological research. It serves as a visual tool for multivariate predictive modeling, enabling clinicians, researchers, and patients to efficiently assess risks, prognoses, or diagnostic outcomes (8, 9). Hence, this study explores DF risk factors in T2DM patients by integrating noninvasive detection of radial pulse wave to construct a predictive nomogram model. The goal is to offer a novel, simple, non-invasive, and cost-effective early warning tool for DF, promote the application of TCM pulse diagnosis in chronic disease management, reduce DF-related disability, and improve patient outcomes.

## 2 Data and methods

### 2.1 Study participants

In this exploratory phase of the study, to mitigate the potential bias introduced by class imbalance in the training data and to enhance the model’s ability to identify DF features while improving training stability and efficiency, we applied dataset balancing techniques during model development. This approach facilitates a more stable preliminary evaluation of model performance in the development stage. To avoid the risk of introducing artifactual noise or undermining the biological fidelity of the data, we refrained from using synthetic oversampling methods (e.g., SMOTE) that generate data points not grounded in actual clinical observations.

Final model validation was conducted using an independent test set that retained the original observed prevalence of the condition. This strategy ensures that performance metrics—such as sensitivity, specificity, and positive predictive value—reflect the model’s generalizability to real-world populations, thereby providing clinically relevant evidence with greater external validity for decision-making.

Due to the retrospective design of this study, DF diagnoses documented in the electronic medical records often lacked standardized staging information, such as Wagner classification. Consequently, stratification of all DF patients according to disease severity was not feasible. The distribution of patients for whom staging information was available is presented in **Supplementary Material 1**.

This retrospective study included 485 patients with T2DM who were admitted to the Departments of Vascular Medicine and Endocrinology at Shanghai Integrated Traditional Chinese and Western Medicine Hospital between July 2023, and April 2025. Participants were included in the study if they were aged 25 years or older, had a confirmed diagnosis of T2DM, and had complete clinical data. Based on the presence of DF, patients were divided into a DF group (*n* = 242) and a T2DM group (*n* = 243).

**Figure 1:**
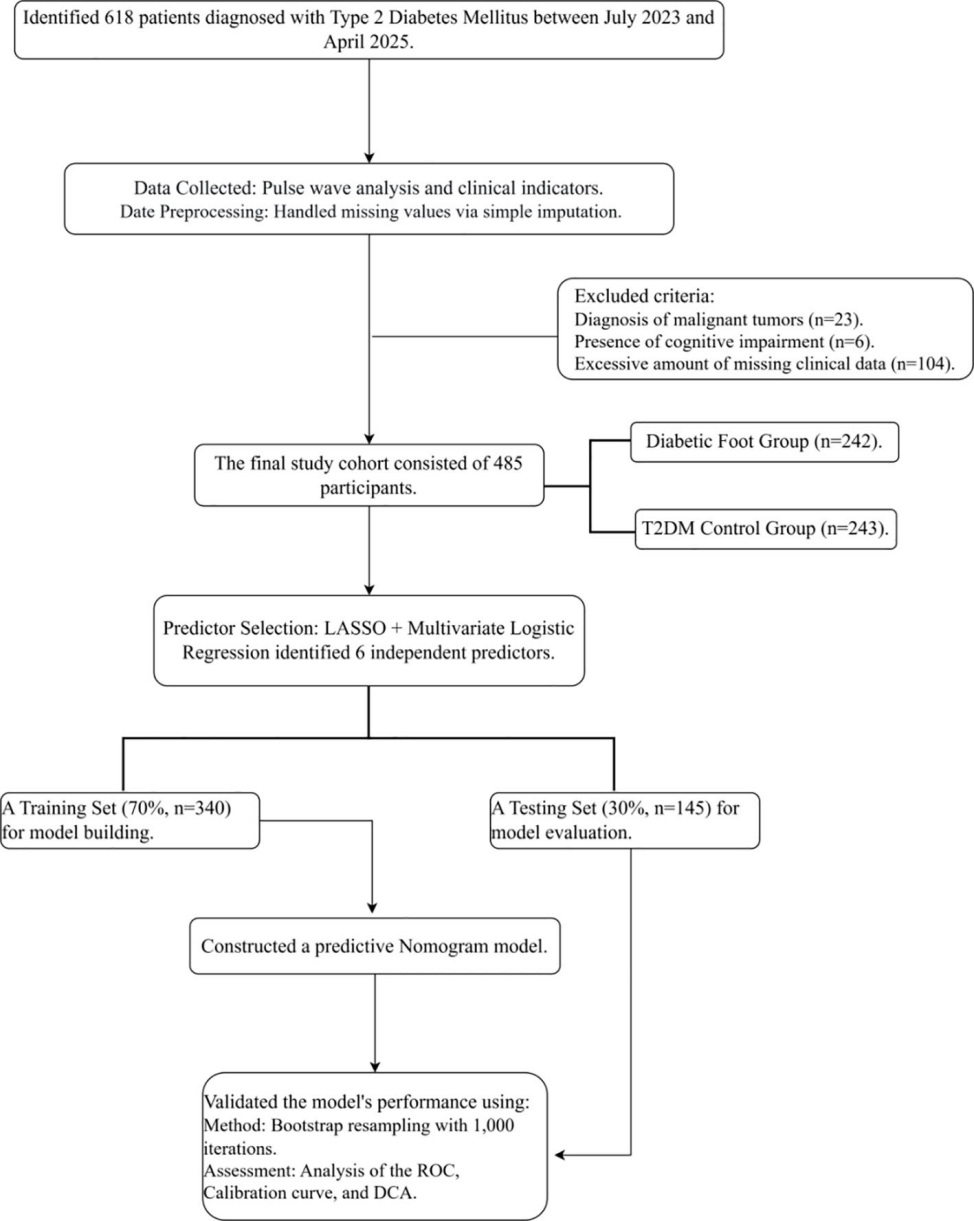
Flowchart of the study.

Diagnostic criteria for T2DM and DF were aligned with the China Guidelines for the Prevention and Treatment of Diabetes (2024 Edition) (10). The detailed criteria with patient inclusion and exclusion criteria are described in **Supplementary Materials 2**.The diagnosis for T2DM was validated through clinical evaluation and laboratory findings, including HbA1c and fasting blood glucose levels, with a documented disease duration of at least one year. The study flowchart is shown in **Figure 1**.

The study was reviewed and approved by the Ethics Committee of Shanghai Integrated Traditional Chinese and Western Medicine Hospital, affiliated with Shanghai University of Traditional Chinese Medicine (Approval No. 2022-004-1). Informed consent is obtained from all participants before data collection.

### 2.2 Measurement indicators

#### 2.2.1 Pulse waveform parameter acquisition

Pulse waveform parameters were collected using the digital pulse analysis system for TCM (11, 12). The standardized procedure was as follows: participants were placed in a sitting or supine position with the forearm naturally extended and maintained at heart level. The wrist was kept straight and relaxed, with the palm facing upward. A pulse pillow was placed under the wrist joint, and a pressure sensor was positioned over the guan region of the cunkou (13) (The cunkou is located at the radial side of the wrist, in the depression between the styloid process of the radius and the radial artery. The guan region is a fixed landmark within the cunkou pulse). The optimal pulse-taking pressure was determined dynamically through a stepwise pressure increase method. Once the optimal pressure was identified, participants were instructed to maintain quiet and steady breathing. Pulse signals were continuously recorded for 60 seconds under this optimal pressure. All pulse wave measurements were conducted by a single operator who had received standardized training, and all assessments were performed between 10:00 AM and 4:00 PM. Patients were examined in the supine position following at least 10 minutes of rest to minimize the influence of diurnal variations, body posture, and recent physical activity.

**Figure 2:**
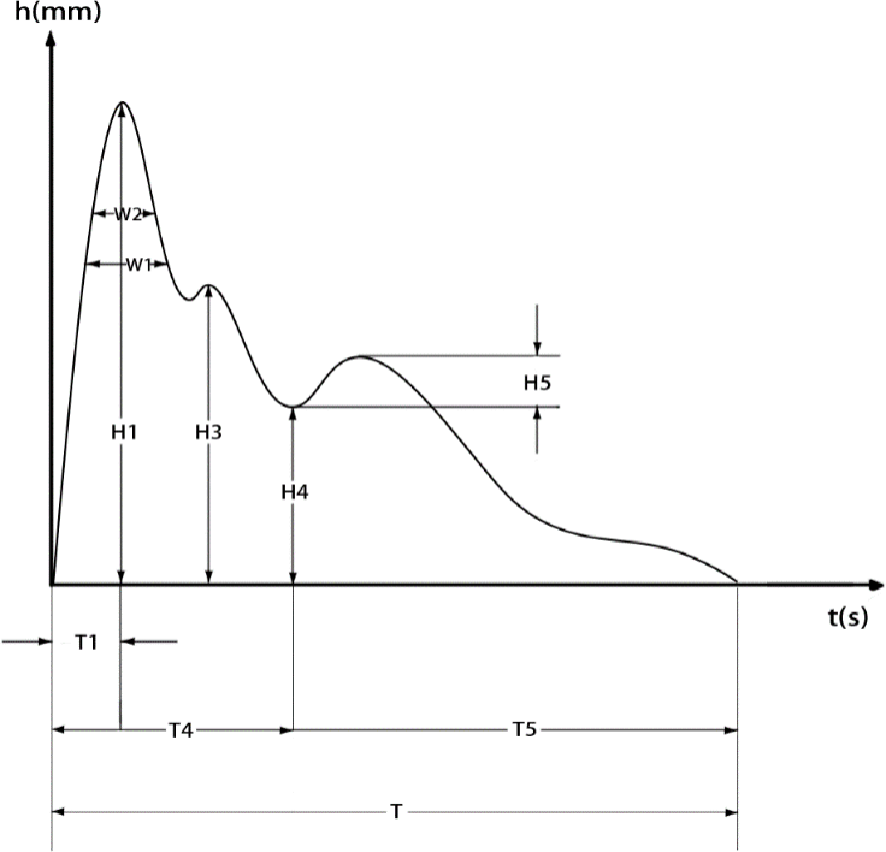
Time-domain parameters of the pulse wave.

The system automatically extracted and analyzed time-domain pulse waveform parameters, including T1, T4, T5, T, H1, H3, H4, H5, W1, W2, As, and Ad. The following parameter ratios were also calculated: H3/H1, H4/H1, H5/H1, T1/T, W1/T, W2/T, and As/Ad (14, 15), as shown in **Figure 2**.

#### 2.2.2 Clinical data

Clinical data were obtained from the hospital’s electronic medical record system. Collected variables included: (1)demographic and clinical characteristics: gender, age, body mass index(BMI), history of smoking and drinking; (2) blood pressure value: systolic blood pressure (SBP), and diastolic blood pressure (DBP), hypertension (HTN); (3)serological markers (preoperative laboratory testing): triglycerides (TG), total cholesterol (TC), low-density lipoprotein (LDL), high-density lipoprotein (HDL), serum creatinine (Cr), blood urea nitrogen (BUN), uric acid (UA), FBG, homocysteine (Hcy), and D-dimer levels.

### 2.3 Statistical methods

To ensure sufficient statistical power and generalization ability, sample size was estimated based on R² shrinkage and the C-statistic for a prediction model with 6 predictors(Variables selected according to the results of the multiple regression analysis: Gender, BMI, HTN, SBP, BUN, H5/H1). The target shrinkage factor was ≥0.90 (16), with an acceptable difference in R² of 0.05. Based on Chinese guidelines and epidemiological data, the prevalence of DF in patients with T2DM is approximately 20%. The target C-statistic and R² were set at 0.63 and 0.11.

All statistical analyses and data visualizations were performed using R (version 4.4.2) and JD_DCPM (V6.11, Jingding Medical Technology Co., Ltd.).

Calculation using the “pmsampsize” package in R indicated a minimum required sample size of 461 participants, including 93 events. This corresponds to 15.37 Events Per Predictor (EPV), which exceeds the recommended threshold of EPV ≥10 (17). The study ultimately enrolled 485 patients with T2DM, of whom 242 were in the DF group, ensuring an adequate number of outcome events. Therefore, the sample size is sufficient for both model development and internal validation.

Data entry was conducted using Microsoft Excel 2021 with double entry and verification. Parameters with more than 10% missing data were excluded. For parameters with less than 10% missing data, simple imputation was performed using R Studio. For continuous variables exhibiting a non-normal distribution, missing values will be imputed using the median of the observed (non-missing) values. For continuous variables with a normal distribution, the mean of the non-missing values will be used for imputation (the number and percentage of missing values for each predictor variable are presented in **Supplementary Table 3**). This imputation procedure is implemented using the ‘transcan’ function from the Hmisc package in RStudio.

Statistical analyses were performed using IBM SPSS 27.0. Normally distributed continuous variables are presented as mean ± standard deviation ( $\bar{\text{x}}$ ± S) and were compared using independent samples t-tests. Non-normally distributed data are reported as median (first quartile, third quartile) [M (Q1, Q3)] and were analyzed using the Mann–Whitney U test. Categorical variables are expressed as frequencies and percentages were compared using the χ² test.

A significance threshold of *α* = 0.05 was set, and *p* < 0.05 was considered statistically significant. Univariate logistic regression was first performed for preliminary variable screening. Variables showing statistical significance were further analyzed using LASSO regression combined with multivariate logistic regression (backward stepwise method) to identify independent risk factors for DF in T2DM patients (*p* < 0.05 considered significant).

### 2.4 Model construction

The R software with the rms package was used to construct the nomogram prediction model based on factors independently associated with DF risk in T2DM patients. The dependent variable was the presence or absence of DF. Data were randomly divided into a training set (70%) for model construction and a test set (30%) for model evaluation. Internal validation of the nomogram was conducted using the Bootstrap method with 1000 repetitions.

### 2.5 Model evaluation

Predictive performance was assessed using Accuracy, Precision, Recall, F1-score, and the Area Under the ROC Curve (AUC). Model calibration was evaluated using the Hosmer–Lemeshow test and calibration curves to assess the consistency between predicted probabilities and observed outcomes. Clinical applicability was analyzed using Decision Curve Analysis (DCA).

## 3 Results

### 3.1 Comparison of clinical data between groups

Comparisons between the T2DM and DF groups showed no significant differences in TC, LDL, UA, or D-dimer levels (*p* > 0.05). The DF group had significantly higher proportions of males, smokers, drinkers, and patients with HTN (coded as 0 = No, 1 = Yes in the table) (*p* < 0.05). Age, TG, Cr, BUN, and Hcy levels were significantly higher in the DF group (*p* < 0.05). BMI, DBP, SBP, and HDL were significantly lower in the DF group (*p* < 0.05) (**Table 1**).

**Table 1:** Comparison of clinical data between T2DM and DF groups.

Variable	T2DM group(*n* = 243)	DF group(*n* = 242)	Statistic	*P*-value
Gender/[*n*(%)]			17.563	<0.001
Male	139 (57.2%)	182 (75.2%)		
Female	104 (42.7%)	60(24.8%)		
Age (years)	68.00 (62, 74)	70.00 (64, 76)	-2.821	0.005
Smoking [*n* (%)]			6.851	0.009
0	156 (64.2%)	127 (52.5%)		
1	87 (34.03%)	115(47.5%)		
Alcohol [*n* (%)]			4.695	0.030
0	196 (80.6%)	175 (72.3%)		
1	47 (19.4%)	67 (27.7%)		
HTN/[*n*(%)]			12.261	<0.01
0	86(35.4%)	51(21.1%)		
1	157(64.6%)	191(78.9%)		
BMI(kg/m²)	24.974[22.758, 27.435]	24.221[21.971, 25.394]	3.319	0.001
SBP(mmHg)	130 (121, 140)	128 (120, 130)	3.757	<0.001
DBP(mmHg)	78 (71, 83)	76[70, 80]	3.460	0.001
TG(mmol/L)	1.29[0.99, 1.87]	1.29[1.05, 1.47]	2.196	0.029
HDL(mmol/L)	1.01[0.92, 1.23]	1.01[0.86, 1.07]	2.757	0.006
Cr(μmol/L)	62[50, 76]	69[57, 94.25]	-3.615	<0.001
BUN(mmol/L)	5.9[4.9, 7.5]	6.7[5.375, 8.8]	-4.333	<0.001
Hcy(μmol/L)	12.9[10.1, 14.4]	12.9[12.6, 15.3]	-2.318	0.021

### 3.2 Comparison of pulse waveform parameters between T2DM groups with and without concurrent DF

When comparing the T2DM and DF groups, no significant differences were observed in H3/H1, T1/T4, T5/T4, W1/T, and W2/T ratios (*p* > 0.05). However, the As/Ad and T1/T ratios were significantly higher in the DF group (*p* < 0.05), while the H4/H1 and H5/H1 ratios were significantly lower (*p* < 0.05), as shown in **Table 2**.

**Table 2:** Comparison of pulse mapping parameters between T2DM and DF groups.

Variable	T2DM group(*n* = 243)	DF group(*n* = 242)	Statistic	*P* -value
As/Ad	3.417 [2.818, 4.49]	4.126 [3.143, 5.171]	-3.375	0.001
H4/H1	0.329 [0.265, 0.392]	0.277 [0.212, 0.355]	4.438	<0.001
H5/H1	0.312 [0.264, 0.359]	0.252 [0.201, 0.315]	6.799	<0.001
T1/T	0.161[0.142,0.178]	0.170[0.146,0.189]	-2.671	0.008

### 3.3 Independent risk factors for DF in T2DM

Univariate logistic regression analysis was conducted on the 17 parameters that showed significant differences between groups. The results indicated that gender, smoking, alcohol consumption, HTN, age, BMI, SBP, DBP, TG, HDL, Cr, BUN, Hcy, and the pulse parameters As/Ad, H4/H1, H5/H1, and T1/T might be influencing factors for DF risk prediction in T2DM (*p* < 0.05) (**Table 3**).

**Table 3:** Results of univariate logistic regression analysis.

Variable	Regression coefficient	Standardized coefficient	OR	95%CI	Wald	*P* -value
Gender	-8.20	0.197	0.441	0299-0.649	17.236	<0.001
Smoking	0.485	0.186	1.624	1.128-2.336	6.815	0.009
Alcohol	0.468	0.217	1.597	1.044-2.442	4.656	0.031
HTN	0.719	0.207	2.051	1.367-3.078	12.052	0.001
Age	0.025	0.009	1.025	1.007-1.043	7.693	0.006
BMI	-0.80	0.025	0.923	0.880-0.969	10.396	0.001
SBP	-0.025	0.007	0.975	0.962-0.989	13.144	<0.001
DBP	-0.036	0.011	0.965	0.945-0.985	11.32	0.001
TG	-0.201	0.097	0.818	0.677-0.988	4.337	0.037
HDL	-0.977	0.363	0.377	0.185-0.767	7.228	0.007
Cr	0.009	0.003	1.009	1.004-1.014	11.158	0.001
BUN	0.139	0.035	1.149	1.073-1.230	15.943	<0.001
Hcy	0.040	0.018	1.041	1.005-1.079	4.955	0.026
As/Ad	0.164	0.051	1.178	1.067-1.302	10.409	0.001
H4/H1	-3.264	0.785	0.038	0.008-0.178	17.274	<0.001
H5/H1	-7.403	1.185	0.001	0.000-0.006	39.024	<0.001
T1/T	8.56	3.242	5217.496	9.068-3002048.589	6.969	0.008

**Figure 3:**
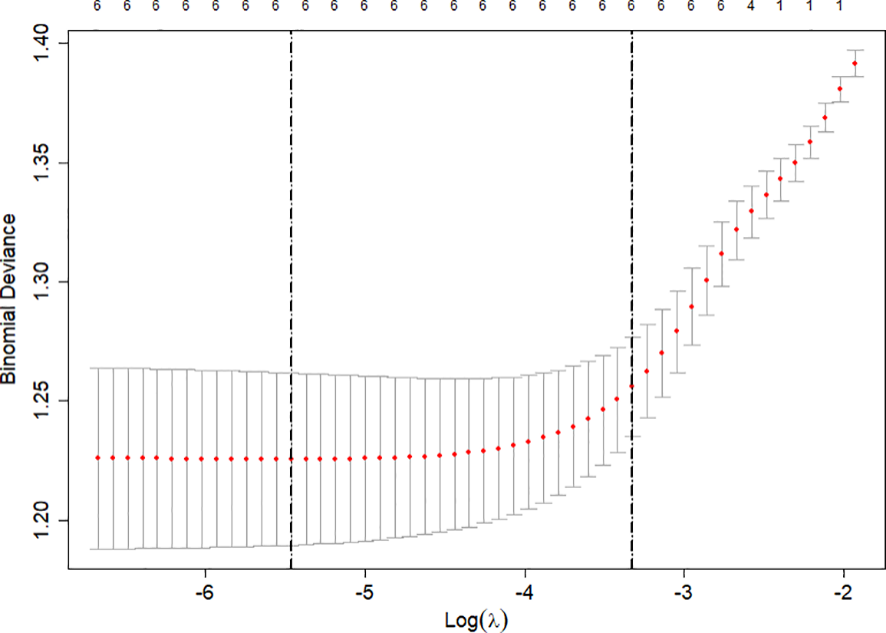
Determination of the optimal value by 10-fold cross-validation in the LASSO regression.

**Figure 4:**
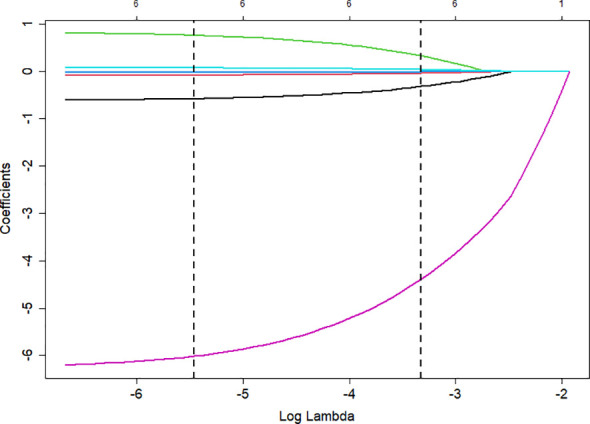
LASSO coefficient path plot.

Variables with statistical significance in the univariate analysis were included in the LASSO regression model for further screening. The analysis results are shown in **Figures 3** and **4**. The optimal lambda (λ) value was determined through 10-fold cross-validation. The two vertical dashed lines represent lambda.min (11 features) and lambda.1se (6 features). To achieve a parsimonious model with stable performance, lambda.1se was selected, resulting in six retained variables: gender, BMI, HTN, SBP, BUN, and H5/H1.

The variables selected by LASSO regression were incorporated into multivariate logistic regression analysis (backward stepwise method). The results identified gender, BMI, HTN, SBP, BUN, and the pulse waveform parameter H5/H1 as independent factors associated with the risk of T2DM complicated by DF (*p* < 0.05) (**Table 4**).

**Table 4:** Results of multivariate logistic regression analysis for DF in T2DM.

Variable	Regression coefficient	Standardized coefficient	OR	95%CI	*P* -value
Gender	-0.691	0.222	0.501	0.324-0.774	0.002
BMI	-0.085	0.029	0.919	0.868-0.972	0.003
HTN	0.909	0.238	2.482	1.556-3.959	<0.001
SBP	-0.028	0.008	0.972	0.957-0.988	<0.001
BUN	0.094	0.037	1.099	1.022-1.182	0.011
H5/H1	-6.252	1.253	0.002	0.001-0.022	<0.001

### 3.4 Establishment and evaluation of the nomogram model

**Figure 5:**
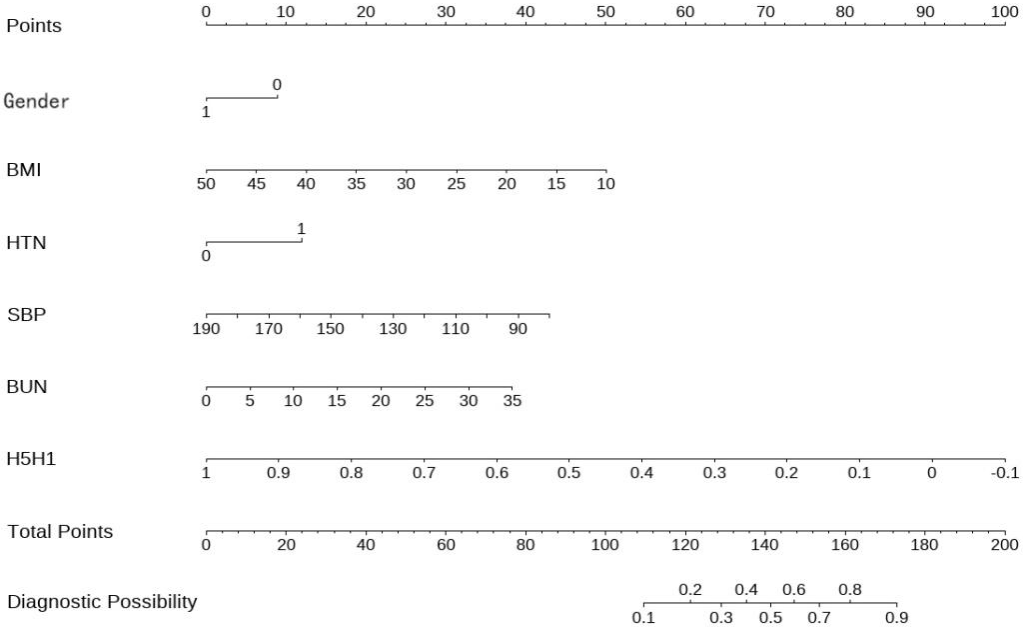
Nomogram model for predicting DF risk in patients with T2DM.

A nomogram model for predicting DF risk in T2DM was developed based on the optimal variables identified in the multivariate logistic regression analysis (**Figure 5**). Points were assigned for gender, BMI, HTN, SBP, BUN, and the pulse parameter H5/H1. The total score was calculated by summing the points, and the corresponding DF risk probability for each T2DM patient was determined based on the total score. The total score ranged from 0 to 200 points: <60 points indicated low risk, 60–120 points indicated medium risk, and >120 points indicated high risk.

**Figure 6:**
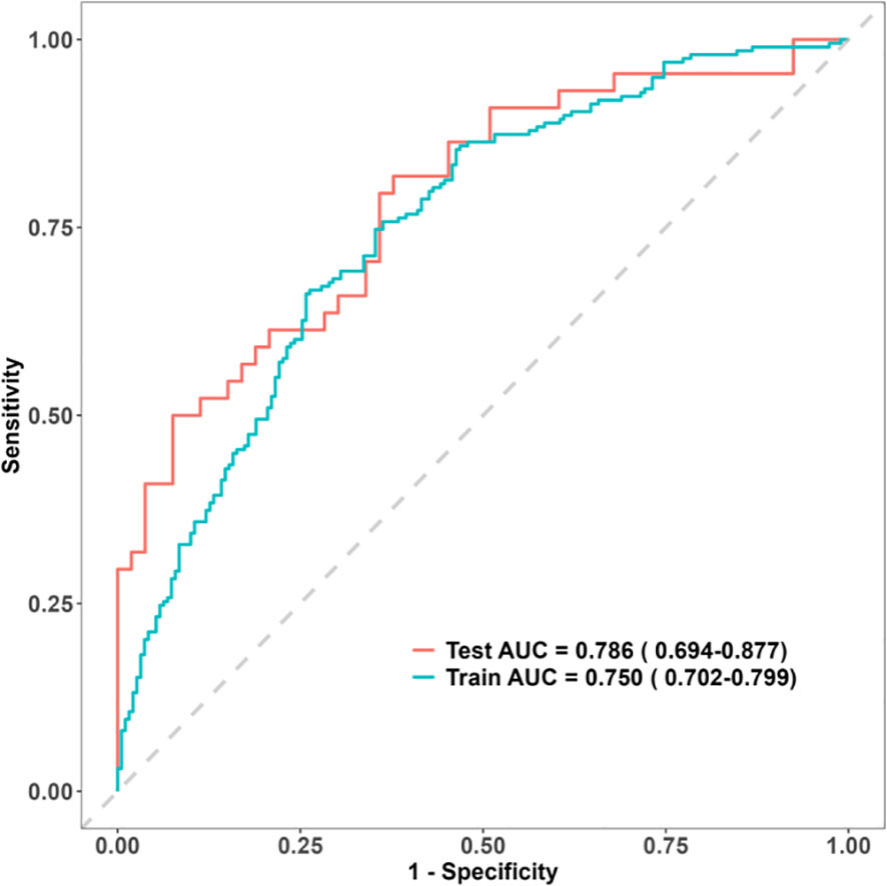
ROC curves of the nomogram model.

The model underwent internal validation using the bootstrap method. Model discrimination was assessed with the ROC curve, calibration was evaluated using the calibration curve, and clinical applicability was examined through DCA. The AUC values for predicting the risk of T2DM complicated with DF were 0.750 [95% CI (0.702, 0.799)] in the training set and 0.786 [95% CI (0.694, 0.877)] in the validation set, demonstrating good discriminative ability of the model. The higher AUC in the validation set compared with the training set indicated that the model was not overfitted and exhibited stable predictive performance for new samples, showing good generalizability. The recall rate in the validation set was 0.7955, indicating that the model identified 79.55% of actual DF cases, effectively reducing the risk of missed diagnoses. These results suggest a satisfactory diagnostic capability, supporting the model’s use for early DF screening (**Figure 6**). The accuracy, precision, recall, and F1-score were 0.701 and 0.7113, 0.7278 and 0.6481, 0.6616 and 0.7955, and 0.6931 and 0.7143 for the training and validation sets, respectively (**Table 5**).

**Table 5:** Evaluation Metrics of the nomogram model.

Dataset	AUC	Accuracy	Precision	Recall	F1-score
Train	0.750	0.701	0.7278	0.6616	0.6931
Test	0.786	0.7113	0.6481	0.7955	0.7143

**Figure 7:**
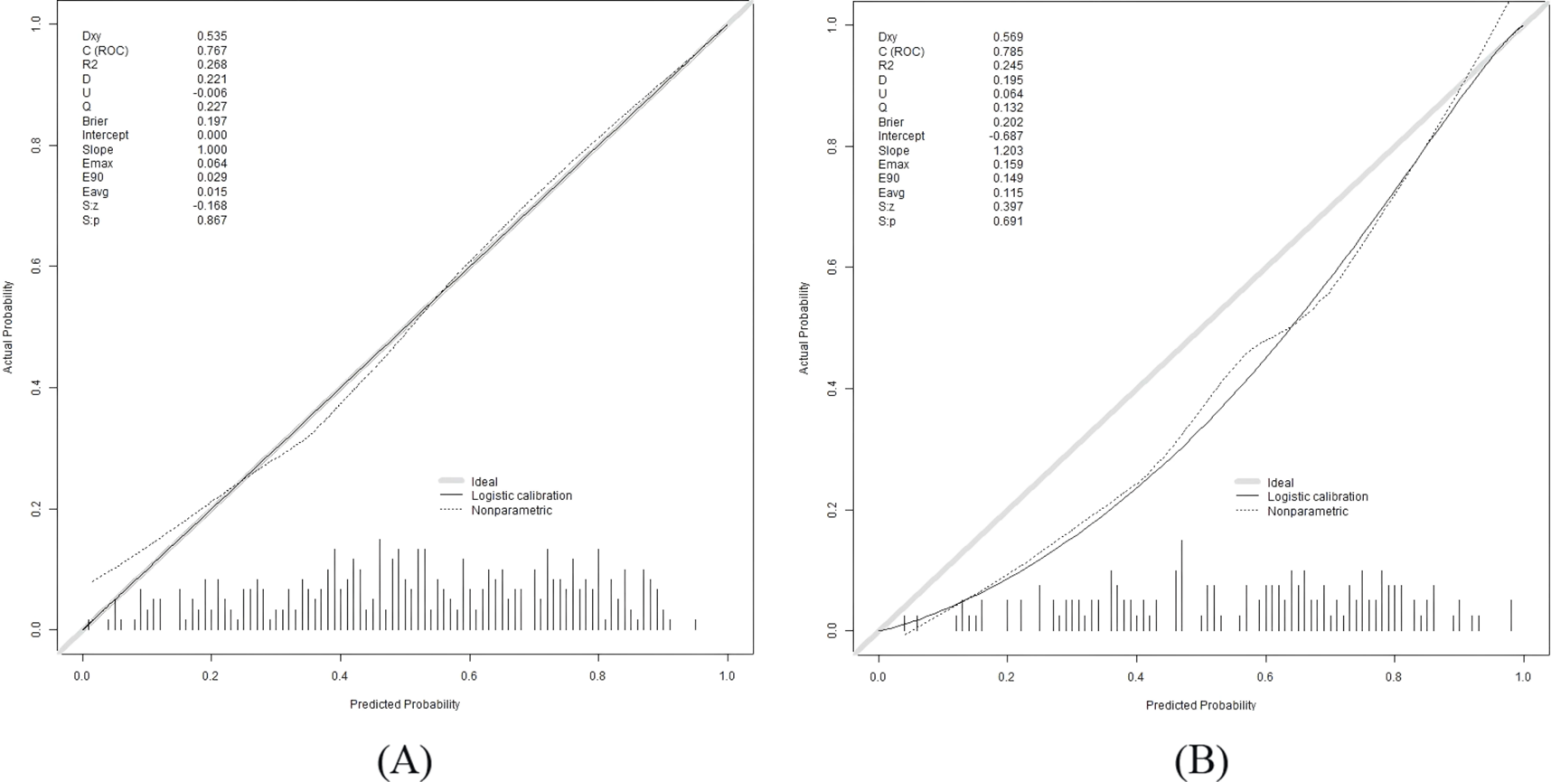
Calibration curves of the nomogram model. **(A)** Train set. **(B)** Test set.

**Figure 8:**
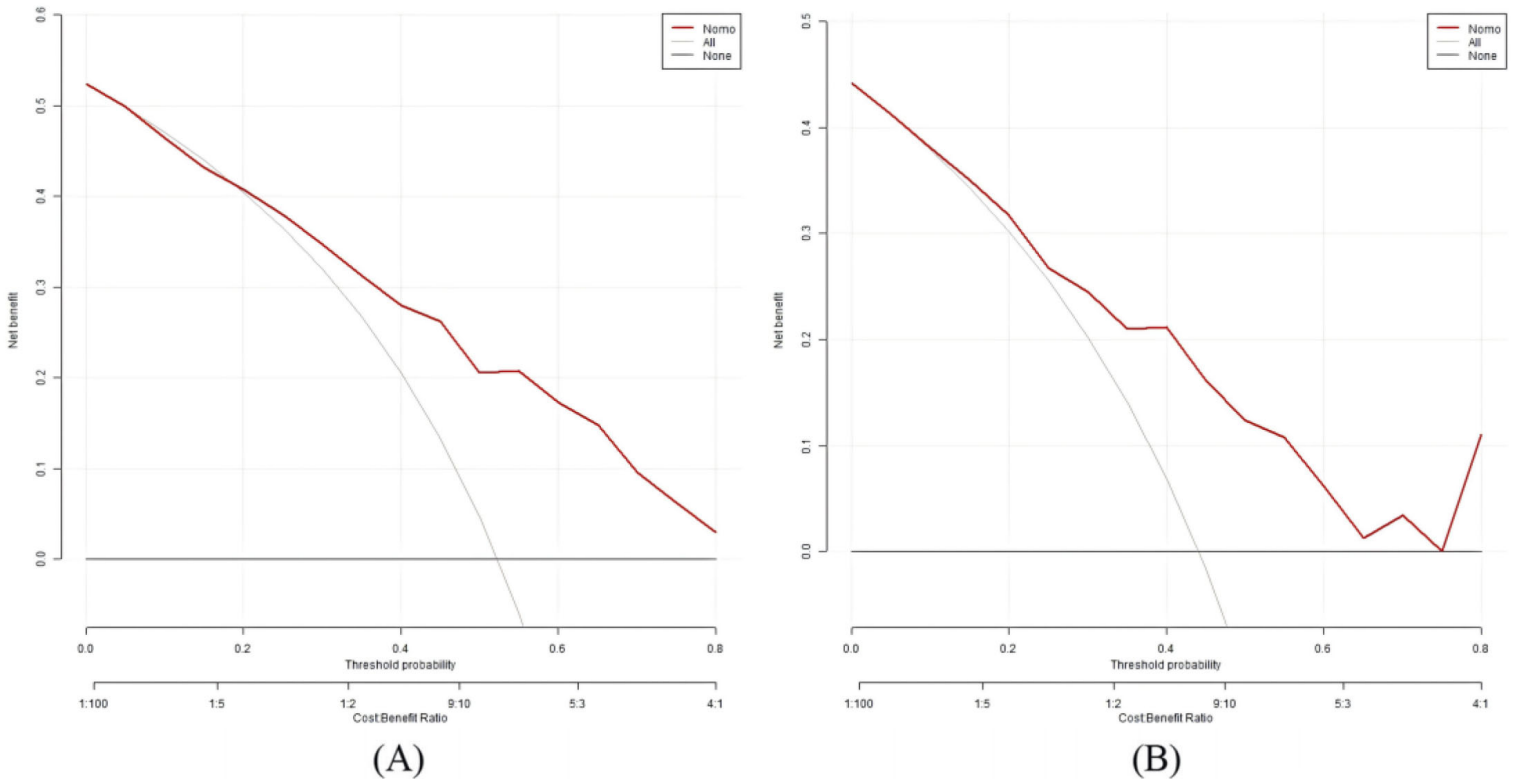
DCA of the nomogram model. **(A)** Train set. **(B)** Test set.

The Hosmer–Lemeshow test result was χ² = 6.1672, *p* = 0.6285. The calibration curves showed close alignment with the ideal curve, indicating good agreement between predicted and observed values and confirming the model’s predictive accuracy (**Figure 7**). The DCA results showed that using this model for DF risk assessment in T2DM patients provided net clinical benefit within a threshold probability range of approximately 0% to 90%, suggesting favorable clinical applicability (**Figure 8**).

## 4 Discussion

Data from the Global Burden of Disease 2021 database show that the disease burden of T2DM in China has continued to increase from 1990 to 2021 and is expected to keep rising in the next five years, presenting major challenges for prevention and control (18). DF is one of the complications that severely threaten the health and survival of DM patients. Early identification and timely intervention are essential for improving the quality of life of DF patients.

Clinical prediction models use mathematical modeling to assign weights to risk factors associated with specific clinical outcomes and visualize the results through tools such as nomograms (19). These models provide clinicians with evidence-based decision support, facilitating the development of individualized treatment strategies through precise risk assessment (20). This study incorporated TCM pulse mapping parameters into a DF risk prediction model for T2DM, aiming to provide a non-invasive, economical, and convenient method for DF risk prediction.

The results of this study indicate that the pulse parameter H5/H1 (OR = 0.002, p < 0.001) is a protective factor against DF risk in T2DM, showing a significant negative effect on risk prediction. This parameter offers a complementary perspective to established DF risk assessment indicators such as the Ankle-Brachial Index (ABI) and Toe-Brachial Index (TBI). While ABI and TBI primarily evaluate structural arterial stenosis or occlusion through direct limb pressure measurements (21), representing static anatomical anatomical assessments, the pulse wave parameter H5/H1 is extracted from the time-domain analysis of the radial artery waveform, reflecting dynamic functional information. Existing research has demonstrated that the radial artery pulse wave plays an important role in predicting adverse cardiovascular and microvascular events in diabetic patients (22). According to hemodynamic principles, H5/H1 is a quantitative indicator of the relative height of the dicrotic wave in the pulse waveform, reflecting large artery compliance and aortic function. H5/H1 decreases when arterial elasticity is reduced or aortic valve insufficiency occurs (23).Therefore, it offers dynamic functional insights into central arterial elasticity and hemodynamics. This represents a shift in assessment perspective—from “peripheral structure” to “central function”—which distinguishes the present study from a TCM viewpoint (24, 25). Existing prediction systems rely predominantly on static indicators, which often fail to capture the critical transition from microcirculatory dysfunction to tissue necrosis (26). In contrast, pulse wave parameters may provide enhanced sensitivity for detecting early functional deterioration within the vascular system. Previous studies have shown that lower extremity atherosclerosis is a common macrovascular complication of T2DM and plays a key role in the pathogenesis of DF (27). These findings are consistent with the pathophysiological mechanism of DM (28), Future studies involving the simultaneous measurement of pulse wave parameters and ABI/TBI will help elucidate the relationship between central arterial function and peripheral arterial structure, thereby establishing a more integrated risk assessment pathway from “functional warning” to “structural diagnosis”.

The results also show that patients with HTN (OR = 2.482) have a 2.482-fold higher risk of developing DF than those without HTN, consistent with previous studies (29). The coexistence of HTN and T2DM is closely associated with DF occurrence, aligning with findings from a multicenter study in Bangladesh (30). However, SBP was negatively correlated with DF risk, which differs from some existing studies. This may be explained by several factors: (1) treatment-related bias—patients in this study likely received antihypertensive therapy, and varying degrees of blood pressure control may have influenced the results; (2) the J-curve phenomenon—when SBP falls below 120 mmHg, toe microcirculation perfusion pressure may decrease, exceeding the lower limit of autoregulation and resulting in lower limb hypoperfusion, thereby increasing DF risk. The optimal blood pressure management strategy for T2DM remains controversial and inconclusive (31). Although elevated blood pressure is a major risk factor for cardiovascular disease in T2DM and blood pressure control can reduce cardiovascular risk, excessive lowering may also lead to adverse outcomes (32). Therefore, individualized blood pressure management is essential for patients with high DF risk, and clinicians should remain vigilant regarding the J-curve risk associated with overly aggressive blood pressure reduction or low-pressure states.

The findings indicate that a higher BMI is associated with a reduced risk of DF, which contrasts with certain prior studies reporting an increased risk of DF with elevated BMI (30). This observation partially supports the so-called “obesity paradox in diabetes” phenomenon (33, 34). However, this association should be interpreted with caution, as BMI does not differentiate between fat mass and lean muscle mass, thereby limiting its objectivity and comprehensiveness as an indicator of obesity. In individuals with long-standing diabetes, sarcopenic obesity—defined by the coexistence of excess adiposity and muscle atrophy—is frequently observed and may mask the true metabolic risk that BMI purports to reflect (35). Indeed, evidence suggests that predicted fat mass demonstrates greater discriminative accuracy than conventional metrics such as BMI and can independently predict the risk of T2MD (36). Furthermore, a linear relationship between predicted body fat percentage and T2DM incidence has been documented (37), indicating that fat accumulation may represent a distinct risk factor independent of BMI. The apparent protective effect of higher BMI observed in our cohort may be attributable to confounding factors or compensatory mechanisms, such as improved nutritional status, enhanced insulin sensitivity, or more intensive medical management among individuals with higher BMI (38).

Furthermore, the heterogeneous effects of weight loss interventions underscore the complexity of the relationship between obesity and clinical outcomes. For instance, bariatric surgery demonstrates more benefits in individuals with early-stage T2DM patients, whereas its efficacy is comparatively limited in patients with advanced complications such as DF (39, 40). Therefore, the associations between BMI and other obesity-related indicators warrant further investigation (41), with particular attention on the potential role of sarcopenic obesity in the pathogenesis of DF. Future multi-center, prospective cohort studies should integrate assessments of muscle mass, inflammatory biomarkers, and metabolomic profiles to comprehensively elucidate the intricate interplay between distinct obesity phenotypes and diabetic complications.

This study also found that male T2DM patients were more likely to develop DF than female patients, which agrees with previous research. This difference may be attributed to the protective effects of estrogen in women, which supports cardiovascular health through multiple mechanisms such as regulating vascular function, protecting the vascular endothelium, and preventing atherosclerosis (42). Combined with gender differences in lifestyle and self-care behaviors, understanding the protective mechanisms of estrogen may provide new insights for developing sex-specific DF prevention strategies.

The model constructed in this study includes six predictive variables (gender, BMI, HTN, SBP, BUN, and pulse parameter H5/H1). The total score is obtained by summing the points for each variable, and the predicted probability of DF occurrence is calculated based on the total score. This study quantified the contribution of each variable to DF risk, with H5/H1 serving as a key predictive variable. Clinicians can use this model to rapidly assess individual DF risk, addressing the issue of equal weighting of indicators in traditional scoring systems and providing a visual and individualized basis for clinical decision-making.

The model demonstrates good diagnostic performance and clinical applicability. It can support early identification of high-risk DF populations in DM, which is important for early intervention and for reducing DF-related disability and mortality rates. The recall of 0.7955 on the test set indicates that approximately 20.45% of DF cases may remain undetected. Therefore, this model should not be used as a substitute for comprehensive clinical evaluation, but rather as an auxiliary screening tool. In practice, the risk threshold could be adjusted to enhance sensitivity and minimize missed diagnoses, albeit at the expense of an increased rate of false positives.

This study has several limitations. First, the single-center, retrospective design may limit the model’s generalizability, and external validation using multicenter data was not conducted. Second, there is currently no standardized protocol for TCM pulse acquisition devices, and intraclass correlation coefficient analysis was not performed to evaluate the reproducibility of pulse wave measurements. Third, the absence of uniform and complete disease severity staging in medical records prevented patient stratification by severity level, which may impair the model’s ability to distinguish risk across clinical subgroups. Fourth, key clinical indicators recommended by the IWGDF guidelines—such as duration of diabetes, ankle–brachial index, and monofilament testing—were not systematically collected. This lack of comprehensive data may reduce the comparability of our model with established clinical assessment pathways, notably, diabetes duration was excluded due to incomplete retrospective data. Future studies should employ multicenter prospective designs, adopt standardized measurement protocols, and incorporate a broader range of clinical and body composition parameters to further validate and refine the predictive performance of the model.

In summary, the nomogram model constructed in this study for predicting DF risk in T2DM demonstrates strong predictive performance. It simplifies the risk assessment process and provides a practical tool for screening high-risk DF populations in primary healthcare settings. Future research could further integrate TCM diagnostic methods with electronic health records, artificial intelligence algorithms, and mobile health applications to establish an integrated “screening–warning–intervention” system for DF prevention and management, combining traditional Chinese and Western medicine approaches.

## 5 Conclusion

This study identified gender, BMI, HTN, SBP, BUN, and the pulse waveform parameter H5/H1 as independent predictors of T2DM patients complicated by DF. Based on these variables, this study developed a nomogram model that demonstrated high discriminative accuracy and clinical utility. By enabling personalized risk stratification, this tool may support more precise clinical decision – making for T2DM and DF. Prospective multicenter validation is warranted to confirm its generalizability and facilitate its integration into clinical practice.

## Data Availability

The raw data supporting the conclusions of this article will be made available by the authors, without undue reservation.
