# Supplementary material for: Development and validation of a nomogram model integrating noninvasive detection of radial pulse wave for predicting diabetic foot risk in type 2 diabetes mellitus
Source: Front Endocrinol (Lausanne). 2026 Feb 12;17:1758234. doi: 10.3389/fendo.2026.1758234 (PMC12935602; doi:10.3389/fendo.2026.1758234)
Supplement: Supplementary file 1 [file DataSheet1.docx]

Supplementary Material 1：

Distribution of Disease Severity Staging among DF： Due to the lack of uniform staging records (e.g., Wagner classification) in some medical records, this section only presents the distribution among DF patients for whom staging information was available.

The severity staging used in this study was based on the Wagner Classification System:

Grade 0: No ulcer, but high-risk foot present.

Grade 1: Superficial ulcer.

Grade 2: Deep ulcer extending to tendon, joint capsule, or bone.

Grade 3: Deep ulcer with abscess, osteomyelitis, or joint sepsis.

Grade 4: Localized gangrene.

Grade 5: Extensive gangrene involving the entire foot.

**Distribution of Staging :**

| Wagner Grade | Number of Patients (*n*) | Percentage (%) |
| --- | --- | --- |
| Grade 0 | 0 | 0 |
| Grade 1 | 0 | 0 |
| Grade 2 | 9 | 3.7% |
| Grade 3 | 26 | 10.7% |
| Grade 4 | 9 | 3.7% |
| Grade 5 | 6 | 2.5% |
| information missing | 192 | 79.3% |

[Supplementary Materials](https://www.frontiersin.org/journals/endocrinology/articles/10.3389/fendo.2025.1657366/full#hsm) 2：

**Inclusion Criterion**: Met the diagnostic criteria for the disease.

T2DM: Diagnoses were made according to the Chinese Guidelines for the Prevention and Treatment of Diabetes (2024 Edition). The diagnostic criteria included fasting blood glucose (FBG), 2-hour blood glucose from an oral glucose tolerance test (OGTT), and glycated hemoglobin (HbA1c). In the absence of typical diabetic symptoms, a diagnosis required at least two abnormal blood glucose values, either from the same or different time points, meeting or exceeding the diagnostic cutoff (excluding random blood glucose).

DF: Diagnoses were made according to the Chinese Guidelines for the Prevention and Treatment of Diabetes (2024 Edition) and met the Wagner classification criteria for grades 1–4 DF as defined in the Chinese Guidelines for the Diagnosis and Treatment of Diabetic Foot .

**Exclusion Criteria:**

Patients meeting any of the following criteria were excluded from the study:1.Those with concurrent malignant tumors.2.Those with severe cognitive impairment or psychiatric disorders.3.Those with extensive missing data, where key clinical variables were missing more than 10% of values, precluding reliable imputation.

Supplementary Table 3

**Missing Data Handling Methods:**

1.Variables with missing values exceeding 10% of the total observations for that variable were excluded from the analysis.For variables with ≤10% missing data, the following imputation methods were applied:

2.Continuous variables (normal distribution): Mean imputation.

3.Continuous variables (non-normal distribution): Median imputation.

**The specific missing data status for key variables is illustrated in the table below:**

| Variable | Number of Missing Cases (n) | Missing Percentage (%) |
| --- | --- | --- |
| BMI | 6 | 1.2% |
| TG | 26 | 5.4% |
| TC | 26 | 5.4% |
| LDL | 41 | 8.5% |
| HDL | 41 | 8.5% |
| Hcy | 19 | 3.9% |
